# Cytological study on Sertoli cells and their interactions with germ cells during annual reproductive cycle in turtle

**DOI:** 10.1002/ece3.2193

**Published:** 2016-05-18

**Authors:** Nisar Ahmed, Huang Yufei, Ping Yang, Waqas Muhammad Yasir, Qian Zhang, Tengfei Liu, Chen Hong, Hu Lisi, Chu Xiaoya, Qiusheng Chen

**Affiliations:** ^1^ Laboratory of Animal Cell Biology and EmbryologyCollege of Veterinary MedicineNanjing Agricultural UniversityNanjing210095China; ^2^ Faculty of Veterinary and Animal SciencesLUAWMSUthal90150Pakistan

**Keywords:** Annual reproductive cycle, Chinese soft‐shelled turtle, morphology, Sertoli cells, vimentin

## Abstract

Sertoli cells (SCs) play a central role in the development of germ cells within functional testes and exhibit varying morphology during spermatogenesis. This present study investigated the seasonal morphological changes in SCs in the reproductive cycle of *Pelodiscus sinensis* by light microscopy, transmission electron microscopy (TEM), and immunohistochemistry. During hibernation period with the quiescent of spermatogenesis, several autophagosomes were observed inside the SCs, the processes of which retracted. In early spermatogenesis, when the germ cells started to proliferate, the SCs contained numerous lipid droplets instead of autophagosomes. In late spermatogenesis, the SCs processes became very thin and contacted several round/elongated spermatids in pockets. At this time, abundant endoplasmic reticulum and numerous mitochondria were present in the SCs. The organization of the tight junctions and the adherens junctions between the SCs and germ cells also changed during the reproductive cycle. Moreover, SCs were involved in the formation of cytoplasmic bridges, phagophores, and exosome secretions during spermatogenesis. Tubulobulbar complexes (TBC) were also developed by SCs around the nucleus of the spermatid at the time of spermiation. Strong, positive expression of vimentin was noted on the SCs during late spermatogenesis compared with the hibernation stage and the early stage of spermatogenesis. These data provide clear cytological evidence about the seasonal changes in SCs, corresponding with their different roles in germ cells within the Chinese soft‐shelled turtle *Pelodiscus sinensis*.

## Introduction

Testes are considered components of both the reproductive and the endocrine systems. The testis parenchyma consists of numerous seminiferous tubules and interstitial tissues. Each seminiferous tubule is composed of germ cells and Sertoli cells (SCs). SCs play a central role in the development of a functional testis and contribute to the expression of the male phenotype (Mackay 2000; Johnson et al. 2008). SCs are irregularly shaped but roughly columnar cells that extend from the basal membrane toward the adluminal compartment in seminiferous tubule in mammals (Russell 1993; Muñoz et al. 2001). The fine cytoplasmic processes of SCs directly contact developing germ cells throughout spermatogenesis (Pescovitz et al. 1994; Griswold 1998).

Sertoli cells play a key role in the regulation of spermatogenesis and the production of spermatozoa. Without the physical and metabolic support of the SCs, germ cells cannot proliferate and survive (Sharpe 1994; Sharpe et al. 2003). SCs are involved in the production of different proteins that regulate or maintain the release of pituitary hormone and also control the mitotic activity of spermatogonia (Johnson et al. 2008). The seminiferous epithelium within the rat testis is divided into basal and adluminal compartments by the blood‐testis barrier (BTB), which is created between adjacent SCs. The BTB forms one of the tightest blood‐tissue barriers in mammals, restricting the diffusion of fluid and preventing the destruction of germ cells by the immune response (Wong and Cheng 2005; Li et al. 2006; Setchell 2008; Tsukita et al. 2008). The cytoskeleton in SC comprises of microtubule, microfilament, and intermediate filament (IFs). Intermediate filaments are composed of vimentin with a molecular weight of 57 kDa. The vimentin is mainly concentrated around the nucleus and plays an important role to maintain cell shape, cell motility, and intracellular trafficking along with other cytoskeleton during spermatogenesis (Kopecky et al. 2005; Lie et al. 2010). Hence, immunohistochemistry (IHC) for vimentin has become the standard marker for SC identification during development, as well as adulthood (Vogl et al. 2008; Weider et al. 2011; Albert et al. 2012).

The morphology of SCs is very complex due to continuous changes in cellular structure and size, and the ultrastructural features differ among species, ages, seasons, and stages of the spermatogenesis (Ghosh et al. 1992; Cudicini et al. 1997; Muñoz et al. 2001). Conversely, with the exception of horses, adult SCs cannot proliferate, and the number of SCs remains constant (Buzzard et al. 2003; Sharpe et al. 2003; Johnson et al. 2008). Therefore, data about SC size and number in seasonal breeders are mysterious. Whereas, several studies have been performed on SCs in mammalian species, very few studies are documented in reptiles (Muñoz et al. 2001).

In several reptilian species, spermatogenesis is independent of male mating behavior (Meisel and Sachs 1994). Interestingly, in the Chinese soft‐shelled turtle (*Pelodiscus sinensis*), spermatogenesis is active through late spring, summer, and fall (May to October), ending in a temporal rather than a spatial pattern, resulting in one massive release of sperm in late October or early November. It remains quiescent (hibernation period) throughout the rest of the year (December to April), so it is a potential model organism with which to determine seasonal effects in China (Zhang et al. 2008, 2015; Chen et al. 2015). To the best of our knowledge, the seasonal changes in SC structure within the Chinese soft‐shelled turtle have not been reported. The principal objectives of the current study were to determine the seasonal effects on morphological changes in SCs, with a focus on the functional aspects of SCs in relation to the germ cells within the testes of the Chinese soft‐shelled turtle *Pelodiscus sinensis*.

## Materials and Methods

### Animals

Fifteen mature, male (3–4 years‐old) *Pelodiscus sinensis* soft‐shelled turtles were purchased from an aqua farm in Nanjing, Jiangsu province of China in January (hibernation period), May (early spermatogenesis) and October (late spermatogenesis), five turtles during each time period. The animals were rendered comatose using intraperitoneally administered sodium pentobarbital (20 mg/animal) and were sacrificed by cervical dislocation. The testes were collected immediately and fixed (details below) for light, electron microscopy, and immunohistochemistry analysis. Sample preparation was conducted according to accepted international standards and was approved by the Ethics Committee for Animal Care and Use by the Science and Technology Agency of Jiangsu Province (SYXK (SU) 2010‐0009).

### Light microscopy

The sample were placed in 10% neutral buffered formalin for fixation overnight and then embedded in paraffin wax, and wax blocks were prepared. Sectioning was performed at 5 *μ*m. These sections were stained with hematoxylin and eosin procedures (Harry's hematoxylin for 2 min and 1% eosin for 30 sec) for light microscopic analysis using an Olympus microscope (BX53), camera (Olympus DP73, Jeol, Tokyo, Japan).

### Transmission electron microscopy

The specimen were cut into small parts (1 mm^3^) and then immersed in 2.5% glutaraldehyde in PBS (4°C, pH 7.4, 0.1 mol/L) for 24 h. Tissue was rinsed in the same PBS and then postfixed for 60 min. at room temperature in the same way using buffered 1% osmium tetroxide (Polysciences Inc. Warrington, PA) and washed in the buffer. The samples were then dehydrated in ascending concentrations of ethyl alcohol, infiltrated with a propylene oxide–Araldite mixture and then embedded in Araldite. The blocks were then sectioned using an ultramicrotome (ReichertJung, Wien, Austria), and the ultrathin sections (50 nm) were mounted on copper coated grids. The pieces were stained with 1% uranyl acetate and Reynold's lead citrate for 20 min. Finally, specimen were examined and photographed using a high resolution digital camera (16 mega pixel) connected to the TEM, Hitachi H‐7650 (Japan).

### Immunohistochemistry

Paraffin sections (6 *μ*m) were placed on glass slides, which are already treated with by poly‐l‐lysine and were stained according to immunohistochemical standard techniques. Briefly, after deparaffinization and washing in phosphate‐buffered saline (PBS), these sections were enclosed with 3% hydrogen peroxide in PBS for 15 min at 37°C in order to block the further activity of endogenous peroxidase. The samples were blocked with 5% bovine serum albumin and incubated with rabbit anti‐vimentin (1:75) antibody (Santa Cruz Biotechnology, Dallas, TX, USA) in a moisture chamber at 4°C for 24 h. After washing, the sections were incubated with biotinylated anti‐rabbit IgG (purchased from Boster Bio‐Technology Co., Ltd, USA) for 1 h at room temperature. The sections were then rehydrated in PBS (PH 7.2), incubated with avidin‐biotinylated peroxidase complex for 45 min at 37°C. After being washed with PBS, peroxidase activity was revealed using DAB (purchased from Boster Bio‐Technology Co., Ltd) according to the instructions of company.

## Results

### Light microscopy

**Figure 1:**
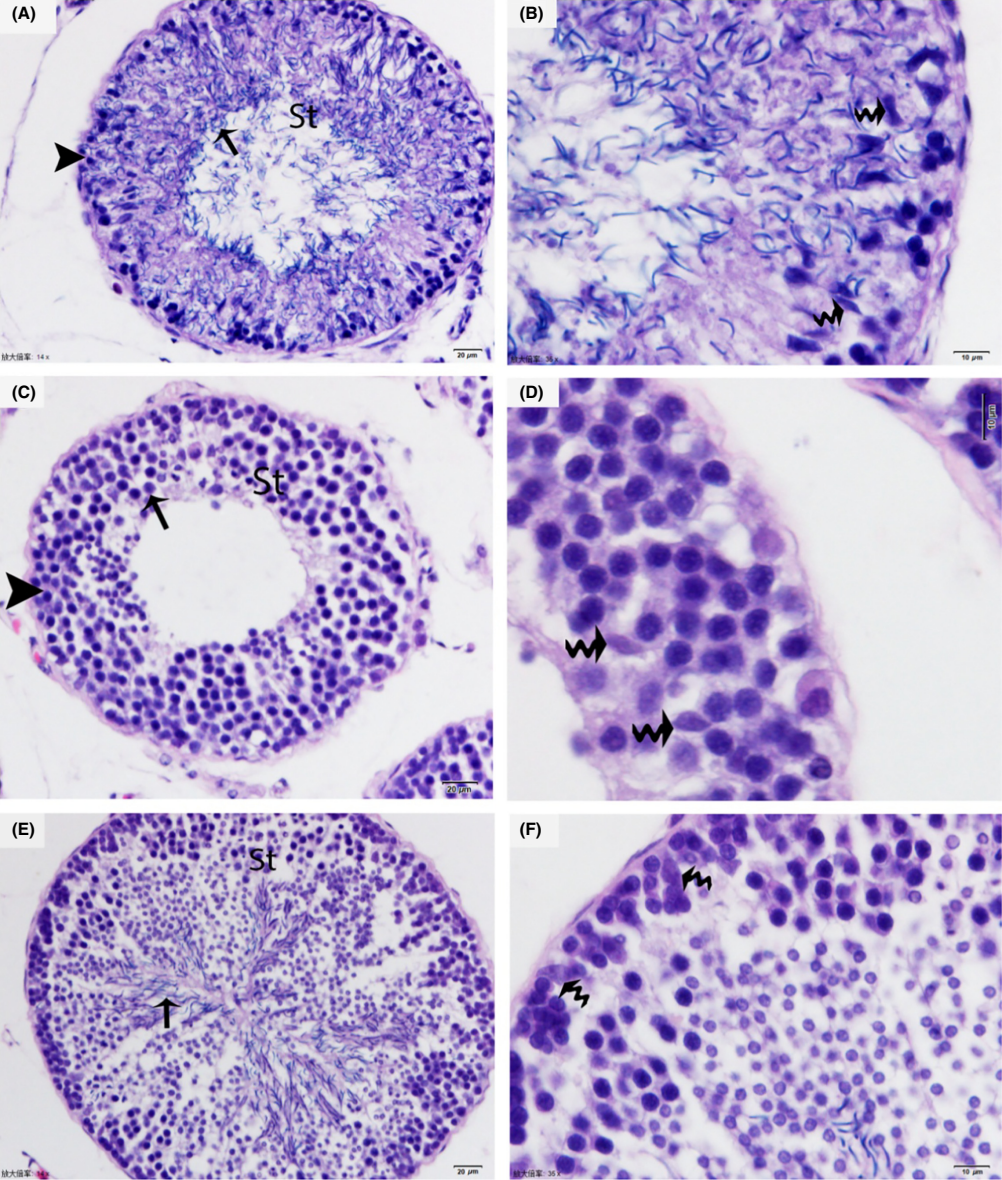
Photograph of H&E staining showing the seasonal changes in histology of *P. sinensis* testes. Spermatogonia (arrowhead), residual elongated spermatid (arrow), and Sertoli cells (curved arrow) are seen in January (A, B). Several layers of spermatogonia (arrowhead), Sertoli cells (curved arrow), and some early primary spermatocytes (arrow) are present in May (C, D). Sertoli cells (curved arrow) organize the germ cells into the sperm column (arrow) in October (E, F). Scale bar = 20 *μ*m (A, C, E) and 10 *μ*m (B, D and F).

In January (hibernation period), SCs with irregular nuclei were observed and were located away from the basal membrane. A few layers of germ cells, typically spermatogonia and residual spermatozoa, were observed in the seminiferous tubules. The adluminal compartment appeared almost clear, and the decreased sperm number was indicative of the testes being cleared into the epididymis (Fig. 1A and B). In May (early spermatogenesis), the nuclei of the SCs appeared flattened or elongated and were located midway between the basal membrane and the lumen. There were several layers of germ cells observed around the SCs, mostly spermatogonia and some early spermatocytes (Fig. 1C and D). In October (late spermatogenesis), the majority of the cell types within the seminiferous tubules were elongated spermatids; the SCs were evenly distributed between them. The SC nuclei were triangular or pear shaped and near the basal membrane. However, the elongated spermatids were arranged in the sperm column around the SCs from the basal to the adluminal compartment (Fig. 1E and F).

### Immunohistochemistry

**Figure 2:**
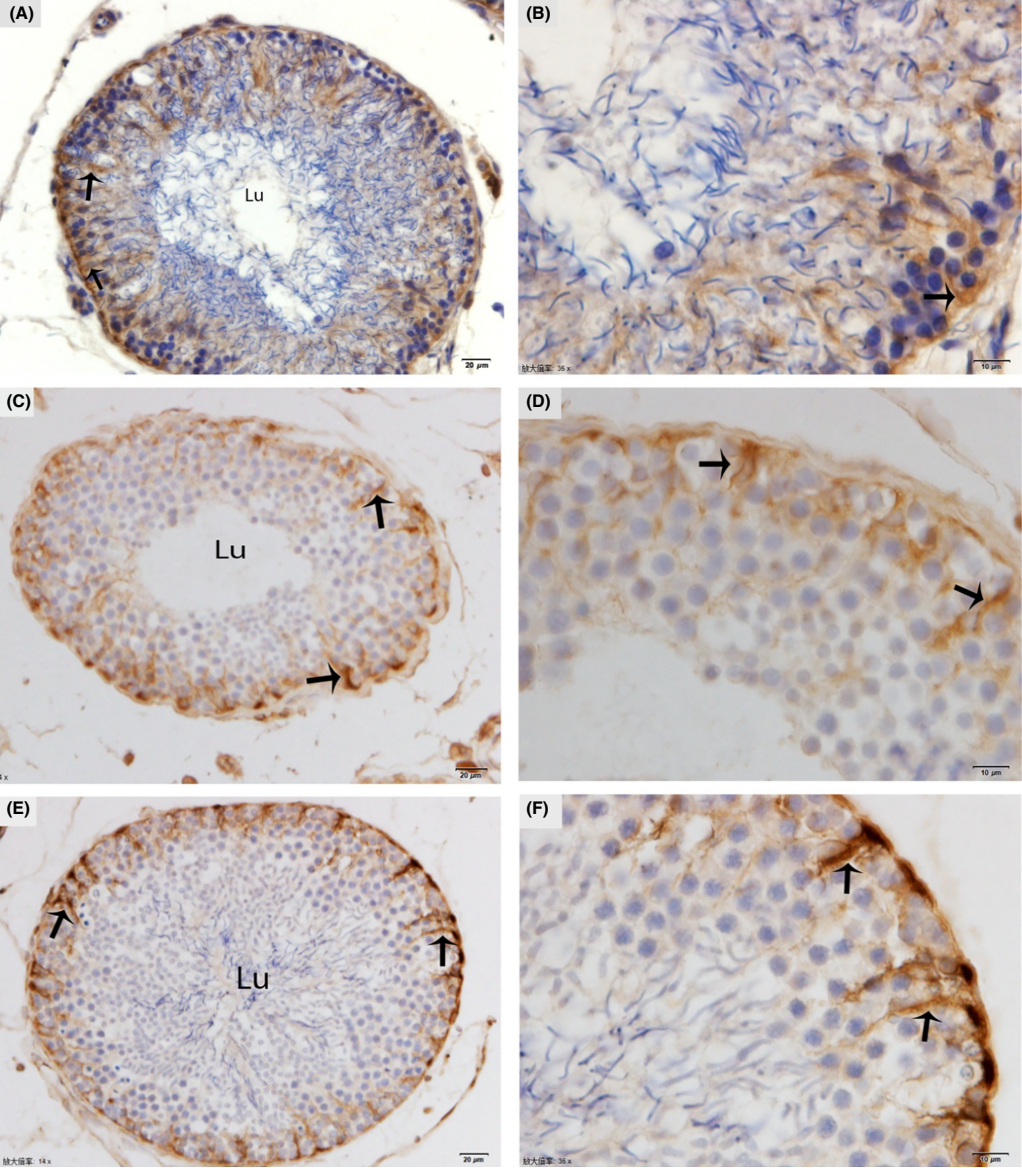
Immunohistochemical localization of vimentin in the testis. The immunoreactivity of Sertoli cells showing weak expression (arrow) in January (A, B), moderate expression (arrow) in May (C, D), and strongly positive expression (arrow) in October (E, F). Lu: lumen. Scale bar = 20 *μ*m (A, C, E) and 10 *μ*m (B, D and F).

In January (hibernation period), weak immunoreactivity of the vimentin antibody was noted in the basal compartment of the SCs, but delaminating from the adluminal compartment of the seminiferous tubule (Fig. 2A and B). In May (early spermatogenesis), moderate immunoreactivity of the vimentin antibody was observed, but only in the basal zone of the SCs, near the basal lamina of the seminiferous epithelium (Fig. 2C and D). In October (late spermatogenesis), strong immunoreactivity of vimentin antibody was noted in the basal portion of the SCs, mainly around the basal lamina (Fig. 2E and F).

### Transmission electron microscopy

**Figure 3:**
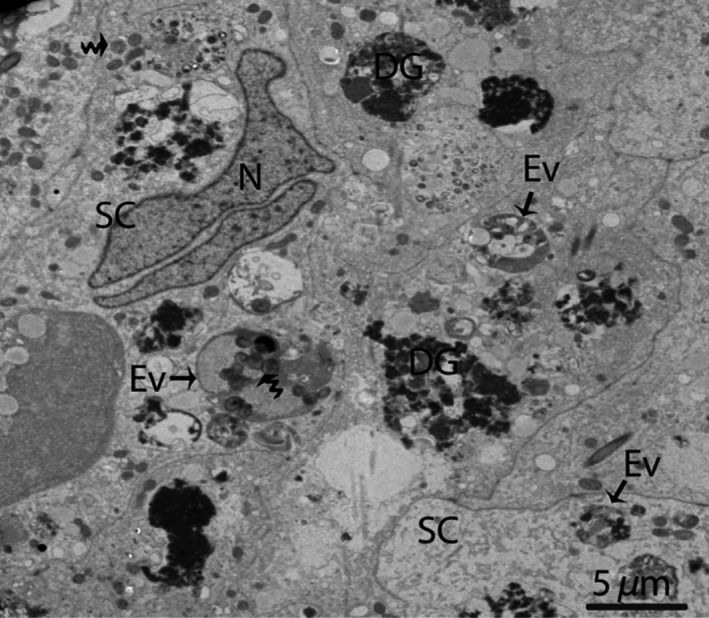
Electron micrograph of entotic vacuoles within Sertoli cells in January. The autophagosomes (curved arrow) and several degenerated germ cells are present inside Sertoli cells. SC, Sertoli cell; N, nucleus; Ev, entotic vacuole; DG, degenerated germ cells. Scale bar = 5 *μ*m.

**Figure 4:**
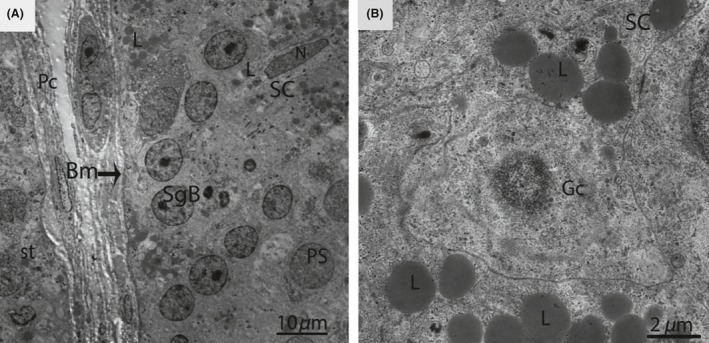
Electron micrograph of seminiferous tubules in May. Sertoli cell exhibits an elongated nucleus (A) and contains several large lipid droplets (B). SC, Sertoli cell; Pc, peritubular cell; Bm, basal membrane; SgB, spermatogonia type B; PS, primary spermatocytes; St, seminiferous tubules; Gc, germ cell; L, lipid droplets; N, nucleus. Scale bar = 10 *μ*m (A) and 2 *μ*m (B).

In January (hibernation period), irregular nuclei and numerous degenerated germ cells were observed in the SCs. The cytoplasm of the SCs contained entotic vacuoles, which contained numerous autophagosomes (Fig. 3). In May (early spermatogenesis), the SCs were located in the basal compartment of the seminiferous tubules. Meanwhile, a deep bordering of developing germ cells, spermatogonia, and primary spermatocytes was noted. The nuclei of the SCs appeared to be elongated, and lipid droplets predominated in the cytoplasm during this period (Fig. 4A and B).

**Figure 5:**
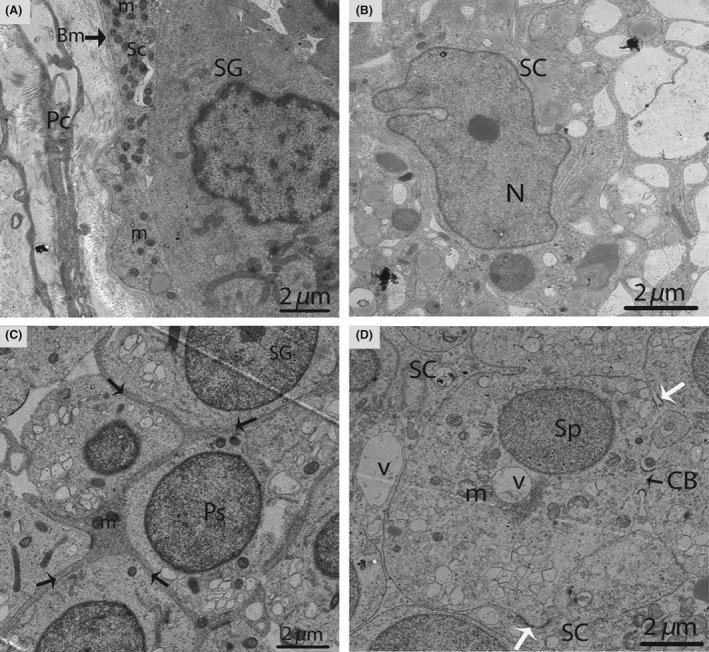
Electron micrograph of seminiferous tubules in October. Sertoli cell exists between the basal membrane and spermatogonia (A). A pear shaped nucleus is observed within the Sertoli cell (B). The thick process of the Sertoli cell (arrow) wraps around the spermatogonia and primary spermatocytes (C). Spermatids are connected by a cytoplasmic bridge and adherens junctions (white arrow) (D). SC, Sertoli cell; Pc, peritubular cell; PS, primary spermatocytes; Sp, spermatid; L, lipid; Bm, basal membrane; SG, spermatogonia; m, mitochondria; CB, cytoplasmic bridge; V, vesicle. Scale bar = 2 *μ*m (A–D).

**Figure 6:**
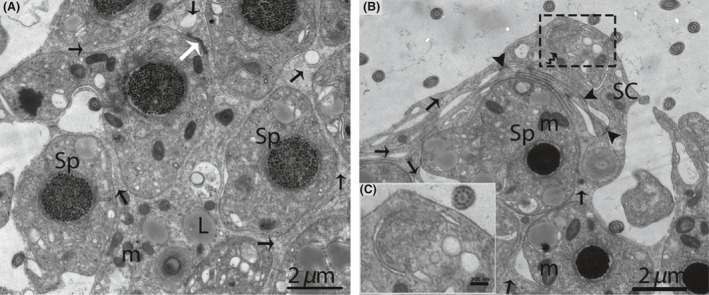
Electron micrograph of the sperm column in October. Sertoli cell (Black arrow) organizes the germ cells into a column of seminiferous epithelia (A). The phagophore (curved arrow) appears within the cytoplasm of a Sertoli cell (B). Illustration indicates the enlarged image of a phagophore (C). Sp, spermatid; SC, Sertoli cell; m, mitochondria; L, lipid; (white arrow) adherens junctions; (arrowhead): tight junctions. Scale bar = 2 *μ*m (A, B) and 200 nm (C).

In October (late spermatogenesis), the SCs were in close proximity to the basal membrane, and irregular invaginations toward the basal membrane were observed (Fig. 5A). The SCs contained pear shaped nuclei, along with a prominent nucleolus (Fig. 5B). Several thick SC processes enveloped the associated germ cells, consequently giving the SCs a three dimensional appearance (Fig. 5C). The SCs processes developed a cytoplasmic bridge between the germ cells (Fig. 5D). Moreover, these extensive cytoplasmic processes enveloped several round/elongated spermatids in pockets, thereby, forming a sperm column from the basal to the adluminal compartment. The cytoplasmic processes of the SCs became thinner as spermatogenesis progressed (Fig. 6A and B).

**Figure 7:**
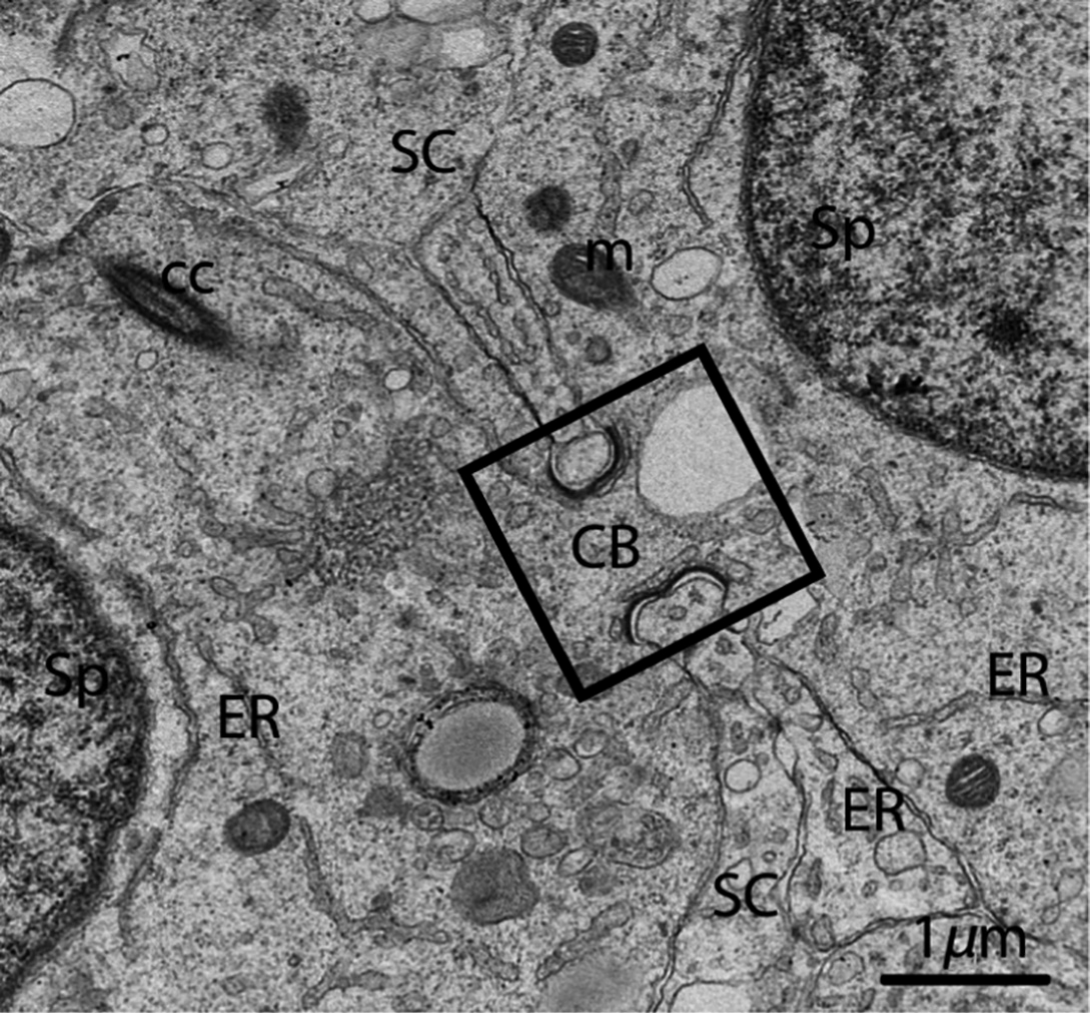
Electron micrograph of a Sertoli cell that exhibits the cytoplasmic bridge (rectangular area) in October. SC, Sertoli cell; Sp, spermatid; CB, cytoplasmic bridge; ER, endoplasmic reticulum; m: mitochondria; CC, centrosome. Scale bar = 1 *μ*m.

**Figure 8:**
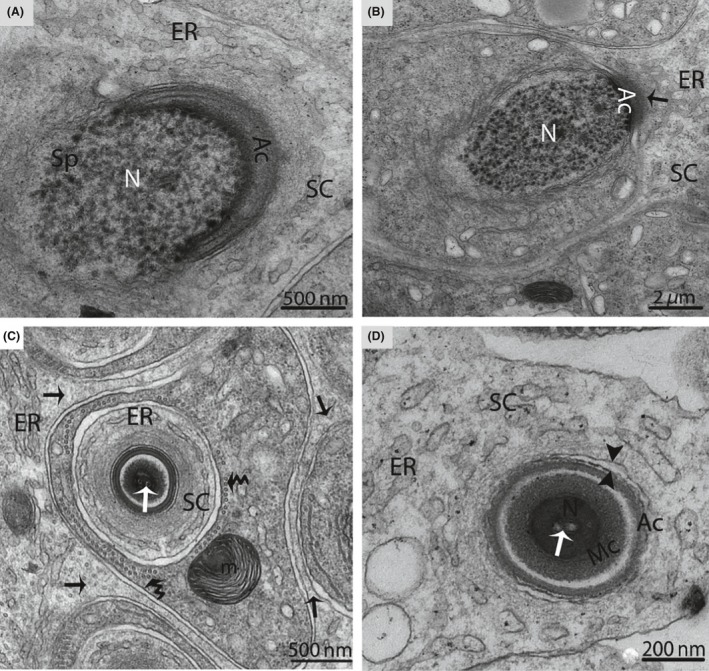
Electron micrograph of a Sertoli cell during spermiogenesis in October. The Sertoli cell (arrow) fully encloses the spermatid during the formation of the acrosomal cap (A). Endoplasmic reticulum secretes a dense material (arrow) near the acrosome (B), and a large number of tubules are observed inside the Sertoli cell (arrow) (C). Opposite arrowheads indicate the cell membrane of the Sertoli cell, which is separated from the spermatid (D). SC, Sertoli cell; Sp, spermatid; Ac, acrosome; N, nucleus; ER, endoplasmic reticulum; m, mitochondria; Mc, manchette; (white arrow): intranuclear canal; (curved arrow): microtubules. Scale bar = 500 nm (A), 2 *μ*m (B), 200 nm (C) and 500 nm (D)**.**

**Figure 9:**
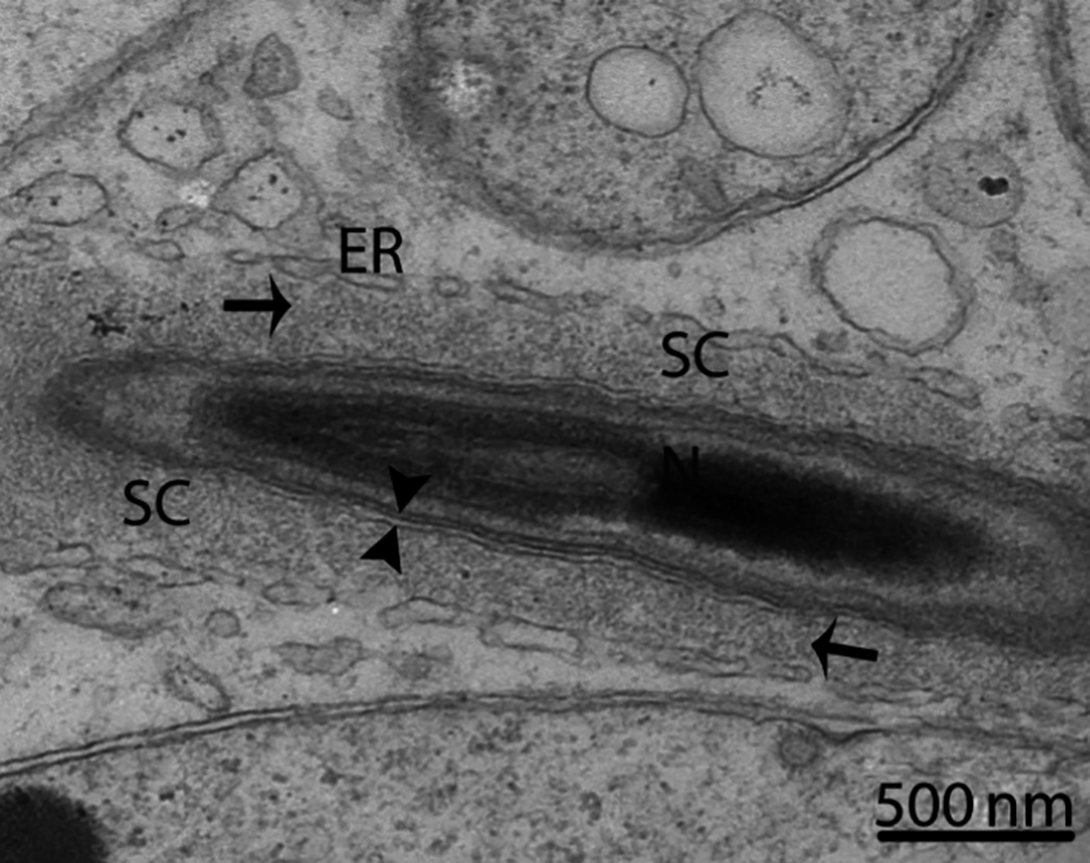
Electron micrograph of a Sertoli cell around the longitudinal section of a spermatid in October. The Sertoli cell contains microfilaments (arrow). SC, Sertoli cell; ER, endoplasmic reticulum; N, nucleus; (opposite arrowhead): cell membrane of a Sertoli cell separated from a spermatid. Scale bar = 500 nm.

**Figure 10:**
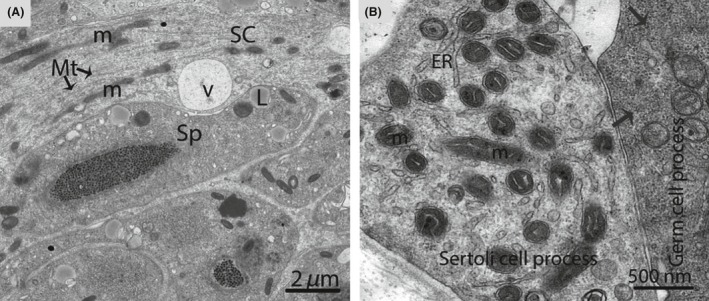
Electron micrograph of a Sertoli cell process in October. Sertoli cells containing several long mitochondria and microtubules (A). The process of the germ cell contained glycogen granules (arrow) (B). SC, Sertoli cell; ER, endoplasmic reticulum; m, mitochondria; Mt, microtubules; V, vesicle; L, lipid droplet. Scale bar = 2 *μ*m (A) and 500 nm (B).

A double membrane, cup‐shaped structure known as the phagophore appeared within the cytoplasm of the SCs. This cup‐shaped structure invigilates the cytoplasm of SCs, inside the double membrane (Fig. 6B). Electron microscopic imaging revealed a unique structure known as the cytoplasmic bridge between the two spermatids, which was composed of SCs. (Fig. 7). The SCs surrounded the head of the spermatid during the development of the acrosomal cap and contained an abundance of endoplasmic reticulum. This endoplasmic reticulum exhibited many tubules and dense material secreted at the site of the acrosomal cap (Fig. 8A–D). An extensive network of microfilaments was observed inside the SCs around the nucleus of the spermatid, and these microfilaments conferred a darker appearance to the SCs around the head of spermatid (Fig. 9). A large number of microtubules were observed within the cytoplasm of the SCs. The SCs contained extremely long mitochondria, which appeared oval, round or elongated in shape. Furthermore, the morphology of some of the mitochondria became onion‐like within the cytoplasm of the SCs (Fig. 10A and B). The endoplasmic reticulum and mitochondria were both observed as predominant organelles in the SC cytoplasm in this period of the reproductive cycle (Figs. 8, 9, 10). In addition, the germ cell processes appeared darker than the SC processes, indicating the excessive deposition of dark granules (glycogen) in the germ cell processes (Fig. 10B).

**Figure 11:**
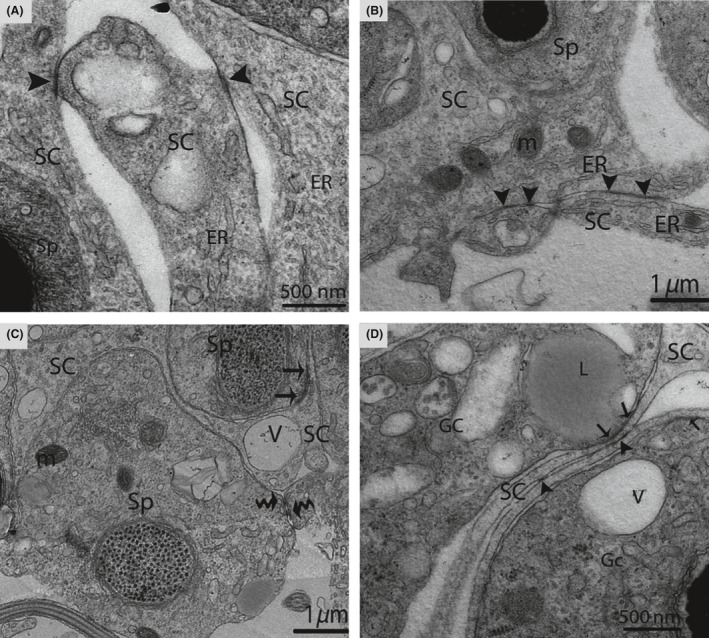
Electron micrograph of the junctional complex in October. Inter‐Sertoli cell tight junctions (arrowhead) (A, B, D), adherens junctions (arrow) (C, D), and desmosome‐like junctions (curved arrow) are detected (D). SC, Sertoli cell; Sp, spermatid; ER, endoplasmic reticulum; V, vesicle; m, mitochondria; Gc, germ cell; L, lipid. Scale bar = 1 *μ*m (B, C), 500 nm (A and D).

**Figure 12:**
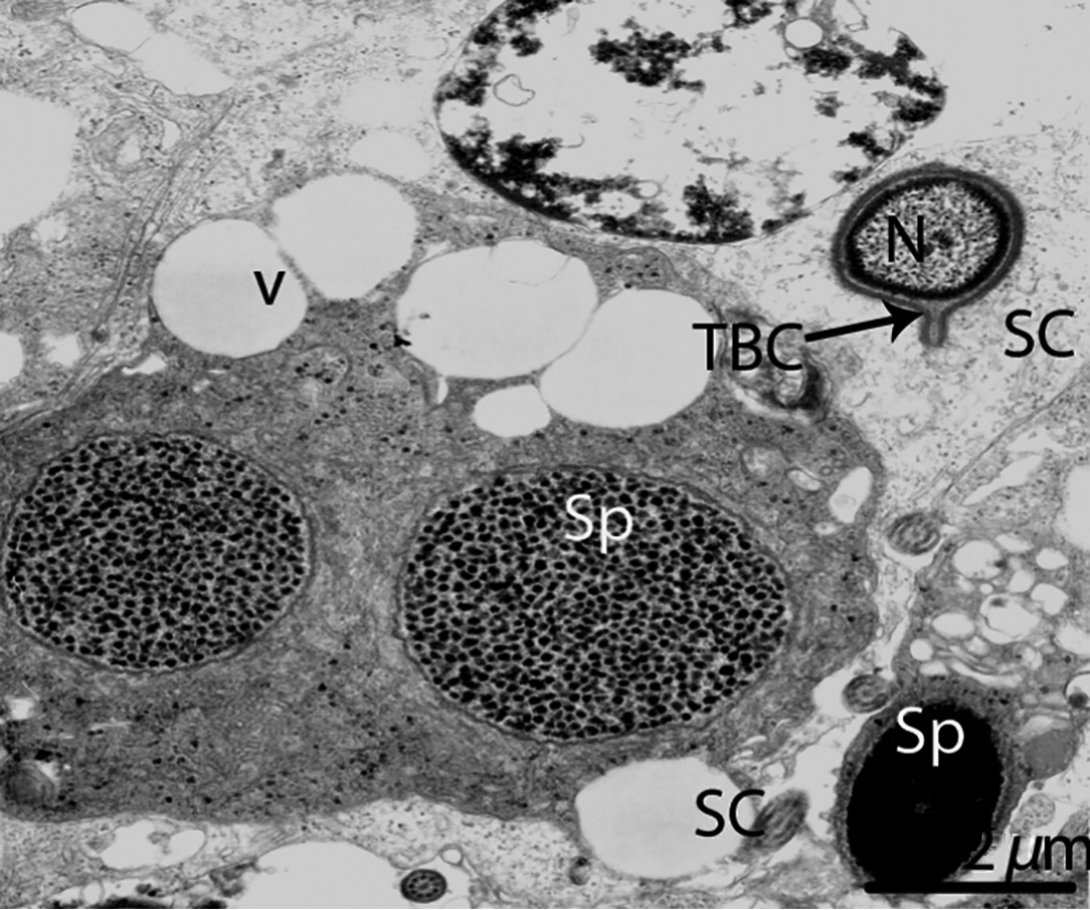
Electron micrograph of the tubulobulbar complex in October. SC, Sertoli cell; TBC, tubulobulbar complex; Sp, spermatid; N, nucleus; V, vesicle. Scale bar = 2 *μ*m.

**Figure 13:**
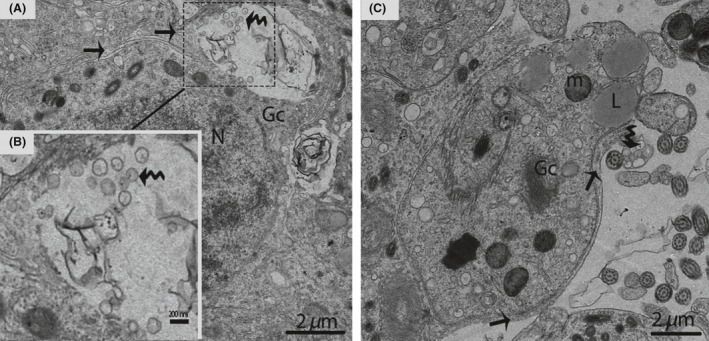
Electron micrograph of exosome secretions in October. The Sertoli cell (arrow) shows the secretion of exosomes (curved arrow) (A). Illustration indicates a magnified view of the exosomes (curved arrow) (B). The exosomes (curved arrow) are enclosed in a membranous structure (C). Gc, germ cell; L, lipid droplet; N, nucleus; m, mitochondria. Scale bar = 2 *μ*m (A), 200 nm (B) and 2 *μ*m (C).

**Figure 14:**
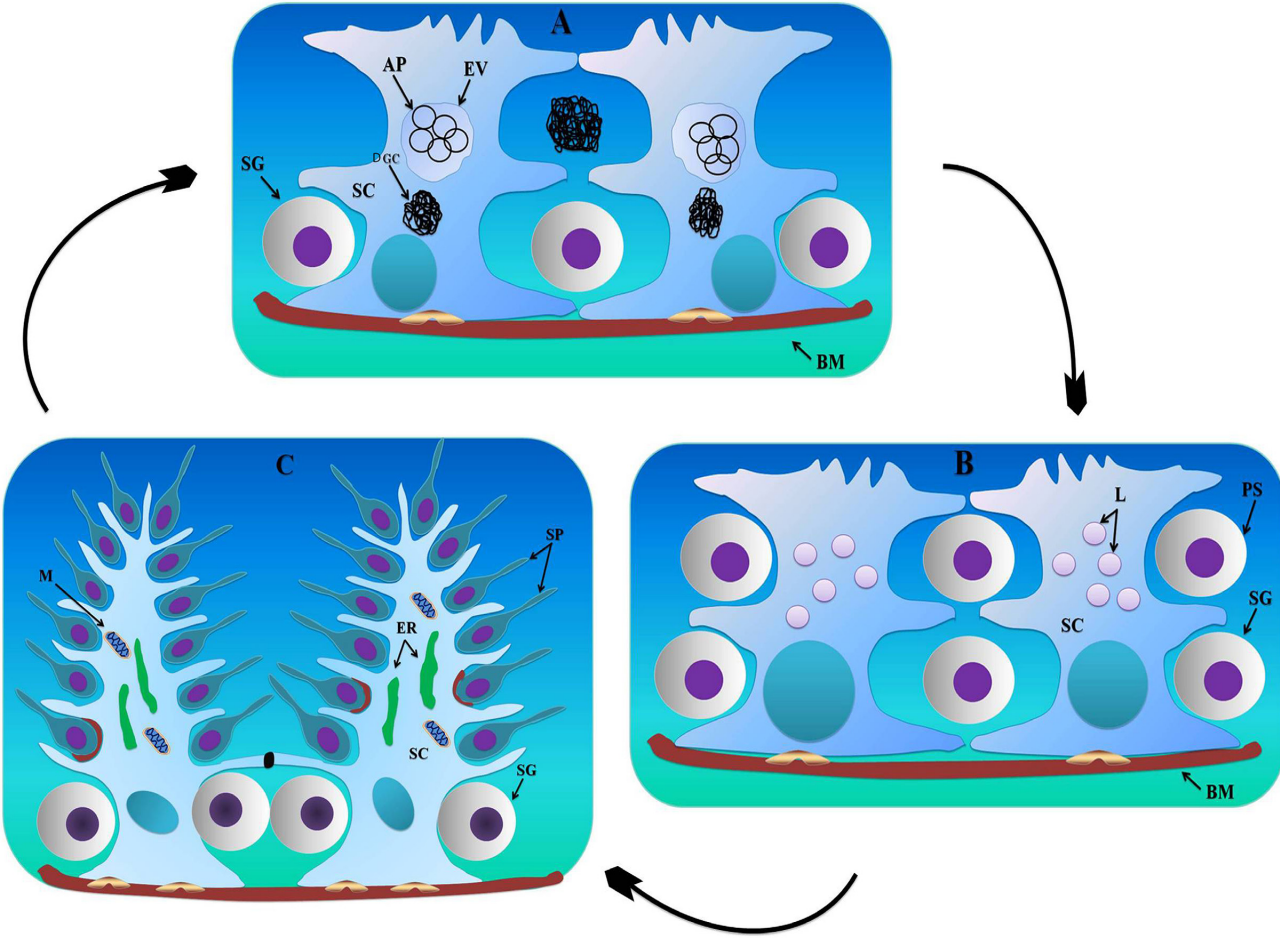
Scheme illustrating the relative changes within the Sertoli cell during the reproductive cycle in the *P. sinensis* testis. In January (hibernation period) the Sertoli cells contain entotic vacuoles (A); during May (early spermatogenesis), they contain numerous lipid droplets (B); and in October (late spermatogenesis), there are abundant endoplasmic reticulum and mitochondria, along with well‐developed processes (C). (black spot): tight junctions; (brown red spot): adherens junctions; (silver yellow spot): hemidesmosomes; SC, Sertoli cell; Bm, basal membrane; SG, spermatogonia; DGC, degenerated germ cells; Ev, entotic vacuole; AP, autophagosomes; Ps, primary spermatocytes; Sp, spermatid; L, lipid droplets; M, mitochondria; ER, endoplasmic reticulum.

Furthermore, the processes of the SCs were interconnected by well‐developed tight junctions between the basal and the adluminal compartment (Figs. 6B, 11A,B and D). Similarly, extensive adherens and desmosome‐like junctions were also detected between the SCs and the germ cells during active spermatogenesis (Figs. 5C, 11C and D). The SCs developed the tubulobulbar complex, the elongated tubular invaginations of the spermatid plasma membrane that project into the SC (Fig. 12). A transmission electron micrograph also provided clear evidence of the secretion of exosomes by SCs in the extracellular compartment around the germ cells (Fig. 13A and B). Figure 14 shows a scheme illustrating the relative changes in the morphology of SCs during different periods of the reproductive cycle in *P. sinensis* testes.

## Discussion

Since the discovery of Sertoli cells (SCs) in the late 1800s, their morphology has been difficult to understand because they continuously change their structure and have three–dimensional contact with developing germ cells (Mruk and Cheng 2004; Hess and França 2005). Cellular function is always closely associated with structure and morphology (Davidson and Mccloskey 2005; Zhang et al. 2015). In this current study, we substantiated for the first time the marked differences in SC function on the basis of morphological evidence in relation to the different stages of germ cell development during the annual reproductive cycle of the Chinese soft‐shelled turtle.

Our IHC result of vimentin in SCs became progressively stronger from January. to May to October., indicating a developing cytoskeleton, which favors increased mechanical force and the formation of a long sperm nucleus. Furthermore, these findings suggest that the intermediate filaments have distinct patterns of distribution during the cyclic process of spermatogenesis and well developed during late spermatogenesis as reported in our TEM images in October (late spermatogenesis) around the nucleus of germ cells. Vimentin filaments also help to maintain the integrity of SC contact with the seminiferous epithelium around spermatogenic cells. Therefore, they play a key role in maturation of spermatogenesis and gap junction intercellular communication (Show et al. 2003; Hejmej et al. 2007; Kotula‐Balak et al. 2009). Moreover, the differential expression of vimentin was reported in equine SCs by Lydka et al.*,* suggested that the Sertoli vimentin filaments are either located around the nucleus and extended to the apical region of SCs in the normal testis and/or collapsed toward the nuclei as in the cryptorchid testes of rat (Wang et al. 2002; Lydka et al. 2011).

During the regression period, the sizes of the SCs and principal organelles were significantly reduced (Muñoz et al. 2001), phagocytosis of residual spermatid bodies and cytoplasm after spermiation by the SCs in mammals was observed (O'Donnell et al. 2011; Dadhich et al. 2013; Seco‐Rovira et al. 2013). Furthermore, the elongated sperm and other residual germ cells were removed by apoptosis, and the remaining of apoptosis was phagocytized by SCs in the Syrian hamster (Wang et al. 2006; Seco‐Rovira et al. 2009, 2014). Our TEM results are further supported by the above findings. We clearly demonstrated the presence of autophagosomes in SCs, indicating the phagocytosis of residual germ cells during the hibernation period. We observed numerous lipid droplets within the cytoplasm of SCs during early spermatogenesis; suggesting a highly nutritive role for SCs with respect to developing germ cells. Normally, drastic increases in lipid droplets within SCs are evidence of spermatogenic arrest or germ cell damage (Hodgson et al. 1979; Bergmann 1987; Ghosh et al. 1992; Wang et al. 2006). Similarly, accumulations of lipid inclusions in viscacha SCs during gonadal recovery have been reported (Muñoz et al. 2001). In contrast, no differences were reported in the amount of lipid droplets in active and inactive SCs within the golden hamster (Hikim et al. 1989).

This present study provides clear evidence that the SCs were well developed, with thin cytoplasmic processes, and formed the sperm column during late spermatogenesis. This illustrates that the SCs stretch their cytoplasm to communicate directly with developing germ cells and provide structural support. Similar findings have been reported in viscacha SCs (Muñoz et al. 2001), and *P. maackii* (Park et al. 2015) throughout the nonbreeding period. In mammalian SCs, abundant RER was observed during active spermatogenesis and correlated with the secretion of proteins (Russell 1993). However, we observed abundant endoplasmic reticulum (ER) around the germ cells, suggesting that SCs play a role in synthesizing proteins, packing and secreting cellular products, and shaping the spermatid during late spermatogenesis. Moreover, we hypothesized that the endoplasmic reticulum is linked with the formation of the autophagosomal membrane because we detected phagophores within the cytoplasm of SCs, cup‐shaped double membrane sacs known as isolation membranes. Isolation membranes increase in size and eventually close to form double‐membrane structures called autophagosomes (Geng et al. 2008; Shibutani and Yoshimori 2014). Some recent studies in mammalian cells found strong evidence about the relationship between the autophagosome formation site and the ER (Axe et al. 2008; Shibutani and Yoshimori 2014). These further support the “ER cradle model,” which states that 70% of the autophagosomes contain ER portions (Hayashi‐Nishino et al. 2009). Additionally, we detected several mitochondria within the SCs, which suggests a high metabolic rate during active spermatogenesis (Russell 1993). We have seen a cytoplasmic bridge between the spermatids that may be involved in the separation of spermatids or in connecting germ cells, as suggested by Johnson et al. (2008).

Our study clearly demonstrates that the junctional complexes became well developed during late spermatogenesis. Inter‐SC tight junctions divide the seminiferous tubules into basal and luminal compartments. Some previous studies reported tight junctions between SCs at the time of active spermatogenesis in vertebrates (Bergmann et al. 1984), viscacha SCs (Muñoz et al. 2001), Djungarian hamster (Tarulli et al. 2008), and *P. maackii* (Park et al. 2015). The above findings suggest that the SCs are involved in the formation of the blood‐testis barrier, which may be associated with spermatogenesis. The blood‐testis barrier protects mature spermatids and spermatozoa from the immune system and controls the movement of small molecules (Li et al. 2006; Tsukita et al. 2008; Du et al. 2013). Additionally, we observed adherens and desmosome‐like junctions between SCs and germ cells. These junctions prevent the sloughing of germ cells from the epithelium and also provide mechanical support throughout spermatogenesis (Kopera et al. 2010).

This current study reveals the existence of tubulobulbar complexes (TBCs) within SCs around mature spermatids. TBCs are fascinating structures appearing mostly in late spermatogenesis, during spermiation (Lee et al. 2006). The basic functions of TBCs include the removal of intercellular ES adhesion junctions, shaping the heads of spermatids and acrosome during spermiation (Guttman et al. 2004; J'Nelle et al. 2009). Interestingly, our TEM findings report for the first time the existence of exosomes within the testes, which may convey a biologic message between SCs and germ cells to regulate spermatogenesis. The microvesicle/exosome‐mediated transfer of transcription factors and nucleic acids between cells or to target cells (Waldenstrom et al. 2012). The nuclei of monkey SCs are located approximately in the middle of the seminiferous tubules, in contrast to various species, including rodents, where the nucleus exists in close proximity to the basement membrane, except during spermiation (Hess 1990). In this current study, the nuclei existed away from the basal membrane during hibernation and early spermatogenesis and were located near the basal membrane during late spermatogenesis. The nucleolus in ruminant SCs is heavily vacuolated or multivesiculated (Pawar and Wrobel 1991), but no vacuoles were observed in the nucleolus of the Chinese soft‐shelled turtle at any stage.

In conclusion, SCs exhibit marked seasonal changes in cytoplasmic and nuclear morphology and size. These morphological changes suggest that the role of SCs changes throughout the annual reproductive cycle, from a phagocytic role during the hibernation period to a highly nutritive role during early spermatogenesis, to a supportive, protective, and cell‐shaping role (for the spermatid) in late spermatogenesis within the Chinese soft‐shelled turtle *Pelodiscus sinensis*.

## Conflict of Interest

The authors have no conflict of interest to declare.
